# Genetic variation of *TLR-4*, *TLR-9 *and *TIRAP *genes in Iranian malaria patients

**DOI:** 10.1186/1475-2875-10-77

**Published:** 2011-04-04

**Authors:** Sedigheh Zakeri, Sakineh Pirahmadi, Akram A Mehrizi, Navid D Djadid

**Affiliations:** 1 Malaria and Vector Research Group (MVRG), Biotechnology Research Center (BRC), Institut Pasteur Iran, Tehran, Iran, Pasteur Avenue, P.O.BOX 1316943551, Tehran, Iran; 2 Biology Department, Khatam University, Tehran, Iran

## Abstract

**Background:**

Toll-like receptors (TLRs) recognize pathogen-associated molecular patterns and their activation leads to the induction of effector genes involving inflammatory cytokines that may have contribute to controlling parasite growth and disease pathogenesis. The current immunogenetic study was designed to analyse the key components of innate immunity, TLRs and TIRAP (Toll-interleukin-1 receptor domain-containing adaptor protein), also known as MAL (MYD88 adaptor-like), in Iranian patients with mild malaria.

**Methods:**

The *tlr*-*4 *(D299G and T399I), *tlr-9 *(T-1486C and T-1237C) and *tirap *(S180L) genes were assessed in 640 Baluchi individuals (320 *Plasmodium falciparum*-infected and 320 non-infected, median age of 28 years) from malaria-endemic regions using polymerase chain reaction-restriction fragment length polymorphism (PCR-RFLP) methods.

**Results:**

Common *tlr-4 *SNPs and promoter SNPs of *tlr-9 *were distributed among *P. falciparum*-infected and non-infected groups (*P *> 0.05) that showed no association of these variants with mild clinical manifestation. The comparison of the *tirap *S180L genotypes between patients with mild malaria and those healthy individuals showed that the frequency of heterozygosity was significantly higher in infected than non-infected individuals (33.8 vs. 25.6; OR, 1.479; 95% CI, 1.051-2.081; *P *= 0.024). The result also revealed a significant association of *tirap *S180L (*P *< 0.05) with development of mild malaria, which is common in Baluchi populations, who are living in malaria hypoendemic region of Iran but not in African populations (0%-6%).

**Conclusion:**

These data point towards the need for addressing the exact role of TLRs in contributing to human genetic factors in malaria susceptibility/resistance/severity within different malaria settings in the world.

## Background

In malaria, clinical manifestations are different among individuals who are infected with *Plasmodium falciparum*, ranging from asymptomatic infection to severe life-threatening forms. The development of severe malaria is due to the marked changes in cytokine expression that results in the individual's immune response to parasites. Indeed, the pathogenesis of severe malaria is complex and so far, the sequence of events leading to severe malaria is not completely known. To understand why some infections lead to severe forms of the disease, research has been focused on identifying parasite virulence phenotypes and the genetic make-up of the host [1].

Toll-like receptors (TLRs) have been defined as pattern-recognition receptors whose function is to recognize pathogen-associated molecular patterns (PAMPs), including microbial pathogens, virulence factors and intracellular protozoan parasites [2-4]. The activation of TLRs leads to the induction of effector genes involving inflammatory cytokines and, as a result, provides links between innate and adaptive immunity [5]. *In vitro *and *in vivo *results showed that TLRs (TLR-2, TLR-4, and TLR-9) are central mediators of pro-inflammatory responses to *Plasmodium *infection [6-12]. The adaptor protein TIRAP (Toll-interleukin-1 receptor domain-containing adaptor protein), also known as MAL (MYD88 adaptor-like), mediates downstream signaling of TLR-2 and TLR-4 inducing pro-inflammatory responses [13].

Single nucleotide polymorphisms (SNPs) have been described for *tlr*-*4*, *tlr-9 *and *tirap *genes that alter susceptibility to infectious and inflammatory diseases [14]. For *tlr*-*4*, the most extensively studied genetic variations are two frequently non-synonymous co-segregating SNPs (D299G/T399I) that change the ligand-binding site of the receptor [15]. *tlr*-*4 *polymorphisms are assumed to be correlated with risk of severe malaria as reported by Mockenhaupt and colleagues [7]. Two common *tlr-9 *promoter SNPs, T-1486C (rs1870884) and T-1237C (rs5743836) are assumed to be associated with susceptibility to asthma [16] and placental malaria [8], respectively. The adaptor protein *tirap *S180L SNP (rs8177374) has been reported to diminish TLR-2 and TLR-4 signaling [3,13] and heterozygosis for this SNP has been claimed to confer protection against mild and severe malaria to an extent comparable to HbAs [17].

Regarding the association between human gene polymorphism and disease, most studies for malaria susceptibility/resistance have been conducted on populations from Africa and south-east Asia. There is no information on malaria-associated gene polymorphisms from Iran, where malaria transmission is hypoendemic and there is no record of severe malaria and death due to this disease is rare. Up to this time, it is difficult to confirm the association between certain *TLR *polymorphisms and malaria susceptibility due to differences in ethnicity, location, environmental factors and exposure to infectious pathogen; hence, conducting different studies on various populations are needed to clarify this issue. Therefore, the current study was designed to look at the polymorphisms in *tlr*-*4 *and *tlr-9 *genes in Baluchi ethnic groups from south-eastern parts of Iran with unstable and hypoendemic malaria transmission. In addition, SNP in *tirap*, which is a common adaptor protein for TLR-2 and TLR-4, was assessed. Finally, the frequencies of the common SNPs, observed in *tlr*-*4, tlr-9 *and *tirap *genes, among Baluchi patients with mild malaria and healthy control individuals were compared. The obtained results might enable us to understand the nature and outcome of the malaria disease in humans, and evaluate novel diagnostic and therapeutic strategies for this disease.

## Methods

### Study areas and population

Malaria is endemic in Sistan and Baluchistan Province (Iran), and the transmission is year-round with two peaks, the first from May to August with *Plasmodium vivax *as the predominant species and the second from October to November when both *P. falciparum *and *P. vivax *infections are usually recorded. In 2008, approximately 11,460 malaria cases were reported in Iran, 10% of which were microscopically diagnosed as *P. falciparum*. Most of the cases (94%) were recorded in the south-eastern provinces and in these regions; there is no report of severe malaria, anaemia or death due to malaria. Moreover, most patients were adults with uncomplicated malaria.

The study population was consisted of 640 Baluchi individuals (420 men and 220 women; median age of 28 years) from malaria endemic region of Sistan and Baluchistan Province. The patients (n = 320) with febrile *P. falciparum *were recruited in an outpatient clinic at primary health centers in Chabahar district during April 2004-November 2009. These patients, who were presented with malaise, fever, or muscular pain and headache, were considered symptomatic and classified as having mild malaria (210 men and 110 women, median age of 28 years) and parasitaemia was ranged 1,000 to 35,000 asexual parasites/mm^3^. All patients who were infected with *P. falciparum *had a fever and the diagnosis was confirmed via microscopy. Healthy Baluchi individuals (n = 320, 205 men and 115 women; median age of 27 years) with no history of febrile or malaria clinical symptoms were included as control. Nested PCR was used to confirm infection and also co-infection with *P. vivax *in both patient and control groups. After obtaining an informed consent from adults or the parents or legal guardians of children, 1 ml of blood was collected from the individuals in vacuum EDTA tubes and stored at -20°C. All individuals with fever and confirmed malaria were treated according to the national guidelines. This study was approved by the Ethical Review Committee of Research in Pasteur Institute of Iran.

### Detection of parasites by nested-PCR assay

Genomic DNA was isolated from whole blood collected in EDTA tubes, by using the commercially available DNA purification kit (Promega, Madison, WI, USA), and kept at -20°C until use. DNA of *Plasmodium *species (*P. vivax *and *P. falciparum*) was detected by nested-PCR amplification of the small sub-unit ribosomal ribonucleic acid (18ssrRNA) genes using the primers and cycling parameters described previously [18]. The amplified products were resolved by 2%-2.5% agarose gel electrophoresis and stained with ethidium bromide for visual detection by ultraviolet trans-illumination.

### Genotyping and SNPs detection by PCR-RFLP

The non-synonymous SNPs of *tlr*-*4 *D299G and T399I, the two promoter SNPs of *tlr-9 *at positions T-1486C and T-1237C as well as *tirap *S180L SNPs were studied. The different SNPs were identified by PCR-RFLP analysis. The primers, PCR cycling conditions and RFLP are shown in Table 1. All amplifications were carried out in a final volume of 25 μL including 1 μL of template genomic DNA. The primers were used at a final concentration of 250 nM and the reaction mixture contained 10 mM Tris-HCl (pH 8.3), 50 mM KCl, 2 mM MgCl_2_, each of the four deoxynucleotide triphosphates at a concentration of 125 μM, and 0.2 U of Taq polymerase (Invitrogen, Carlsbad, CA). The DNA fragments, obtained following PCR amplification or RFLP analysis, were electrophoresed on 2% to 3% agarose gels (Invitrogen, Carlsbad, CA), respectively.

**Table 1:** Primers and profiles used for PCR-RFLP of the *tlr*-*4*, *tlr-9 *and *tirap *genes

			Temperature °C/time (min)						
						
Genes	Primer	Sequence	A	E	D	C	Product size (bp)	Restriction Enzyme	Cut Product Size (bp)
	**299TLR4F**	ATACTTAGACTACTACCTCCATG	**56** **(1)**	**72** **(1)**	**94** **(1)**	**35**	**213**	***NcoI *(Fermentase)**	**G: 19 + 194**
tlr-4*	**299TLR4R**	TTGTTGGAAGTGAAAGTAAG							
	
	**399TLR4F**	TGTTATCAAAGTGATTTTGGGAGAA	**54** **(1)**	**65** **(1)**	**94** **(1)**	**35**	**185**	***Hinf I *(Fermentase)**	**I: 162 + 23**
	**399TLR4R**	AGGTAAATGAGGTTTCTGAGTGATAGG							

	**-1237TLR9F**	TTCATTCAGCCTTCACTCAG							**T: 264+145+126+23**
***tlr-9***	**-1237TLR9R**	TCAAAGCCACAGTCCACAG	**64** **(1)**	**72** **(1)**	**94** **(1)**	**30**	**558**	***BslI *(Fermentase)**	**C: 264+115+126+ 30+23**
	
	**-1486TLR9F**	TTCATTCAGCCTTCACTCAG	**64** **(1)**	**72** **(1)**	**94** **(1)**	**30**	**558**	***AflII *(Fermentase)**	**T: 413 + 145**
	**-1486TLR9R**	TCAAAGCCACAGTCCACAG							

	**180 ***TIRAP ***F**	AGTGCTGTACCATC**GA**CCTGCTG							
tirap **	**180 ***TIRAP ***R**	TTCCCCTTCTCCCTCCTGTAGTAG	**60** **(1)**	**72** **(1)**	**94** **(1)**	**35**	**161**	***Eam1105I *(Fermentase)**	**S: 141 + 20**

All primers described in this study were designed in our laboratory (GenBank accession no. AF177765.1 for *tlr*-*4*; NW-001838877.2 for *tlr-9 *and NT-033899.7 for *tirap*) except 299TLR4F primer that was described previously [26]. The bold and underline nucleotides located in the 3' of forward primers indicate a mutation, and create a restriction site accordingly.

**A**: Annealing, **E**: Extension, **D**: Denaturation, **C**: No. of cycles

* *tlr*-*4 *(gat) D299G (ggt)

* *tlr*-*4 *(acc) T399I (atc)

** *tirap *(tcg) S180L (ttg)

### DNA sequence analysis

To verify the results obtained by RFLP, all PCR products were sequenced for *tlr*-*4, tlr-9 *and *tirap *genes using the primers described in Table 1. Therefore, the amplified fragments were gel-purified using the QIAquick Gel Extraction kit (Qiagen, Hilden, Germany) following the manufacturer's instructions. Direct sequencing of the DNA fragments was performed in both directions for each PCR product using the dideoxy chain termination procedure (Chemistry V3.1, Applied Biosystems) and also the 3730XL DNA analyser (Applied Biosystems) by MilleGen sequencing service (Labege, France).

### Statistical analysis

The sample size was calculated using OpenEpi software [19], with 80% power and 95% confidence level. Allele and genotype frequencies were calculated for different genes by direct counting. The comparison of the allele and genotype frequencies of the study groups was determined by *X*^*2 *^test using SPSS for windows (version 16.0) and *P *< 0.05 was considered to be significant. To determine if genetic variation in *tlr*-*4*, *tlr-9 *and *tirap *genes were associated with risk of developing malaria, odds ratios (OR) and 95% confidence intervals (CIs) were calculated. The Hardy-Weinberg Equilibrium (HWE) was performed by comparing the observed numbers of different genotypes with those expected under the HWE for the estimated genotype frequencies [20] and *P *> 0.05 was in HWE. Genotypic deviations from HWE were assessed using Pearson's chi-square (*X*^*2*^) statistical test.

## Results

Based on nested-PCR results, a total of 320 patients with mild malaria were shown to be infected with *P. falciparum, as a *mono-infection, and none of the healthy control individuals had either *P. falciparum *or *P. vivax *infections.

### *tlr-4, tlr-9 *and *tirap *polymorphisms

Overall, *P. falciparum*-infected (n = 320) and non-infected (n = 320) Baluchi individuals were successfully analysed for the *tlr*-*4*, *tlr-9 *and *tirap *SNPs by PCR-RFLP methods and the sequencing data confirmed RFLP results. To determine whether there was any frequency difference in the allele (Table 2) and genotype (Table 3) prevalence for each SNP, a comparison was made between the two studied groups. All examined SNPs were presented in studied populations at different frequencies. The frequencies for two polymorphic alleles in *tlr*-*4 *(D299G and T399I) and *tlr-9 *(T-1237C) were less than 10% and those for polymorphic alleles in *tlr-9 *(T-1486C) and *tirap *(S180L) were greater than 10% (Table 2) in infected patients. Furthermore, there were no significant differences in all examined SNPs frequencies among Baluchi patients with mild malaria and related case controls (*P *> 0.05, Table 2).

**Table 2:** Allele frequencies for the *tlr*-*4*, *tlr-9 *and *tirap *polymorphisms in *P. falciparum*-infected and non-infected subjects

		Allele frequency				
			
Gene (**Polymorphism)**		Non-infected		Infected		*P *value
			
		W	M	W	M	
***tlr-4***	**D299G**	0.948	0.052	0.923	0.077	0.068
	
	**T399I**	0.921	0.079	0.923	0.077	0.835

***tlr-9***	**-1486**	0.651	0.349	0.646	0.354	0.815
	
	**-1237**	0.898	0.102	0.923	0.077	0.116

***tirap***	**S180L**	0.862	0.138	0.825	0.175	0.065

**W = **wild allele

**M = **mutant allele

**Table 3:** Genotype frequencies for the *tlr*-*4*, *tlr-9 *and *tirap *polymorphisms in *P. falciparum*-infected and non-infected subjects

		Genotype Frequency							
					
Gene (**Polymorphism**)		Observed			expected		*P* (**HWE, *X***^***2***^)	OR (95% CI)	*P* value
					
		Genotype	Non-infected No (%)	Infected No (%)	Non-infected No (%)	Infected No (%)			
		W (D/D)	287 (89.7)	276 (86.3)	288 (90)	273 (85.3)		0.721 (0.446-1.166)	0.181
	**D299G**	H (D/G)	33 (10.3)	39 (12.1)	31 (9.7)	45 (14.1)	NI = 0.33 I = 0.013	1.161 (0.709-1.899)	0.553
***tlr-4***		M (GG)	-	5 (1.6)	1 (0.3)	2 (0.6)		-	-
	
		W (T/T)	270 (84.4)	271 (84.7)	272 (85)	273 (85.3)		1.024 (0.667-1.572)	0.913
	**T399I**	H (T/I)	50 (15.6)	49 (15.3)	46 (14.4)	45 (14.1)	NI = 0.125 I = 0.138	0.976 (0.636-1.499)	0.913
		M (I/I)	-	-	2 (0.6)	2 (0.6)		-	-
	
		W (TT)	130 (40.6)	142 (44.4)	136 (42.5)	133 (41.6)		1.166 (0.852-1.596)	0.337
	**T-1486C**	H (TC)	157 (49.1)	130 (40.6)	145 (45.3)	147 (45.9)	NI = 0.149 I = 0.058	**0.710 (0.520-0.971)**	**0.032**
***tlr-9***		M (CC)	33 (10.3)	48 (15)	39 (12.2)	40 (12.5)		1.535 (0.956-2.464)	0.075
	
		W (TT)	270 (84.4)	276 (86.2)	258 (80.6)	273 (85.3)		1.162 (0.749-1.801)	0.503
	**T-1237C**	H (TC)	35 (10.9)	39 (12.2)	59 (18.4)	45 (14.1)	NI < 0.0001 I = 0.0135	1.130 (0.696-1.836)	0.621
		M (CC)	15 (4.7)	5 (1.6)	3 (1)	2 (0.6)		**0.323 (0.116- 0.899)**	**0.023**

		W (S/S)	235 (73.4)	210 (65.6)	238 (74.4)	218 (68.1)		**0.691 (0.492-0.969)**	**0.032**
***tirap***	**S180L**	H (S/L)	82 (25.6)	108 (33.8)	76 (23.7)	92 (28.8)	NI = 0.150 I = 0.0025	**1.479 (1.051-2.081)**	**0.024**
		M (L/L)	3 (1)	2 (0.6)	6 (1.9)	10 (3.1)		0.665 (0.11-4.004)	0.653

**NI**: non-infected

**I**: *P. falciparum-*infected

**HWE**: Hardy-Weinberg Equilibrium (*P *< 0.05)

**W**: Wild type allele, **H**: Heterozygote allele, **M**: Mutant type allele

**OR**: Odds ratios

**CI**: Confidence interval

### Frequencies of *tlr-4, tlr-9 *and *tirap *genotypes

The three genotypes of *tlr*-*4 *D299G were present in Baluchi infected individuals, but *tlr*-*4 *G299G homozygosity was absent in Baluchi healthy individuals (Table 3) that might suggest an association of this genotype with mild malaria clinical symptoms (1.6%, *P *= 0.025). In addition, two genotypes of *tlr*-*4 *T399T and *tlr*-*4 *T399I were observed among cases and controls with no significant difference (*P *> 0.05). *tlr*-*4 *I399I homozygous was absent in both studied groups (Table 3). The comparison of the *tlr-9 *T-1237C and T-1486C genotypes between patients with mild malaria and those healthy individuals showed that the three *tlr-9 *T-1237C and T-1486C genotypes were present among both studied groups (Table 3). Heterozygosity for *tlr-9 *T-1486C was more prevalent among case controls (49.1%) than among malaria patients (40.6%, Table 3). *tlr-9 *C-1486C homozygote was also found in both *P. falciparum-*infected (15%) and non-infected cases (10.3%) with no statistically significant (*P *> 0.05). Furthermore, *tlr-9 *T-1237C frequency was low in the cases (1.6%) and controls (4.7%). The comparison of the *tirap *S180L genotypes between patients with mild malaria and those healthy individuals showed that the frequency of heterozygosity was significantly higher in infected than non-infected individuals (33.8 vs. 25.6; OR, 1.479; 95% CI, 1.051-2.081; *P *= 0.024) (Table 3).

## Discussion

Severe malaria accounts for many of deaths due to the infection with *P. falciparum*. Areas of intense malaria transmission such as African countries tend to have a greater proportion of severe cases, whereas the opposite is seen in areas of lower transmission [21]. Additionally, in some other malaria endemic regions of the world, including Iran, the severe cases are very rare. This, in turn, raises the question of why small subset of *P. falciparum-*infected individuals develops the severe and complicated disease but, others develop a mild and uncomplicated disease or remain asymptomatic. To verify and provide further data on this issue, different studies showed the contribution of host genetic factors to the severe outcome following infection [22] and since then, several genes have been shown to be involved in protection or susceptibility to severe malaria including TLRs that play an important role in innate immunity [23].

Mockenhaupt and co-workers [7] investigated *tlr-2*, *tlr*-*4 *and *tlr-9 *polymorphisms in African children. They found that a common *tlr*-*4 *D299G increases the risk of severe paediatric malaria without affecting on the risk of infection. However, in the present study, 10% of the participants in both groups had less than 15 years old with no sign of severe disease. In addition, in both studied groups, the frequency of *tlr*-*4 *D299G was low and similar. Therefore, this result suggested that more studies on different populations from malaria endemic regions with various endemicity are needed to draw final conclusion on the role of *tlr*-*4 *D299G and its association with malaria manifestation. On the other hand, study by Mockenhaupt and colleagues [7] showed that *tlr*-*4 *T399I predisposes to severe malaria with symptoms such as severe anaemia and respiratory distress. This suggests that *tlr*-*4 *might be involved in *P. falciparum *recognition and host responses in humans, and that *tlr*-*4 *could contribute to the control of the infection. However, in this study, *tlr*-*4 *T399I genotype has been observed in malaria patients and healthy individuals with no statistically significant that showed no association of this genotype with mild clinical manifestation.

In the next step, the frequency of promoter polymorphisms of *tlr-*9 was assessed in target population. *tlr-*9 T-1486C heterozygote and homozygote were found in both *P. falciparum-*infected and non-infected cases, with no association with mild malaria, which was in line with other findings [7,24]. Furthermore, *tlr-*9 T-1237C frequency was low in the cases and controls with no association with mild malaria. To date, the functional roles of this promoter SNP and of T-1486C are unclear, but our data argues against a major role of these polymorphisms in malaria manifestations.

Study by Khor and colleagues [17] in Gambia, Kenya and Vietnam revealed that *tirap *S180L heterozygosity was associated with a significant protective effect against both mild and severe malaria. In addition, study by Leoratti and co-workers [24] in Brazilian populations showed that no association of this genotype was found with mild malaria. In contrast, in the present study, there was a high frequency of heterozygosity (*tirap *S180L) in Baluchi infected individuals than controls (33.8% vs. 25.6%). The comparison of healthy and infected individuals showed that the risk of mild malaria was increased 1.5-fold in individuals with *tirap *S180L (OR, 1.479; 95% CI, 1.051-2.081, *P *= 0.024). It is worth mentioning that the frequency of this heterozygosity was higher among Iranian infected patients with *P. falciparum *(33.8%), who are living in malaria hypoendemic areas (present study), than Ghanan, Gambian, Kenyan and Vietnamese populations (0%-3.5%) with reported severe cases of malaria [25,17]. In fact, the presence of the high frequency of *tirap *S180L variant in Iranian malaria patients with mild malaria might support the role of this SNP in protection against severe malaria by intermediate levels of pathway activation and balanced inflammatory response (by attenuates TLR-2 and TLR-4 signaling pathways).

Moreover, homozygous *tirap *L180L was found in both infected and non-infected Baluchi individuals (0.6% vs. 1%), which was not reported in African and Vietnamese populations, where malaria is highly endemic. The rarity of the mutant allele in African populations might be related with high mortality rate in these populations due to malaria, thus this may strongly reduce the frequency of *tirap *L180L genotype in these regions. This homozygousity suggested to be a risk factor for several diseases, including malaria [25], which is in contrast with the present finding (OR, 0.665; 95% CI, 0.11-4.004; *P *= 0.653). The specific role of *tirap *L180L on malaria disease processes needs further investigation in different ethnic groups.

Overall, wild-type homozygosity for all three examined genes was predominant among patients with mild malaria and healthy control groups. Statistical analysis also showed that all of SNPs were in HWE in the control group except for *tlr-*9 T-1237C. In addition, the observed genotype frequencies in the patients with mild malaria were significantly different from the expected frequencies for *tlr*-*4 *D299G, *tlr-*9 T-1237C and *tirap *S180L SNPs, which are deviated from HWE. This could be explained by genetic drift, non-random mating or an indication of selection acting on specific genotype and at the present, there is no any evidence to prove any of them in this population.

## Conclusion

In summary, in the current immunogenetic study, it seems that the role of TLR polymorphisms is varying in different ethnic groups from different malaria endemicity. High frequency of heterozygous *tirap *S180L in malaria infected individuals point to the role of this genotype as a risk factor for the development of mild malaria, but protect from severe malaria. Comparing these data with previously reported results from other malaria endemic regions [17,25] suggests a need for addressing the exact role of *TLRs *and TIRAP in contributing to human genetic factors in malaria susceptibility/resistance/severity within different malaria transmission settings and manifestation pattern in the world.

## Competing interests

The authors declare that they have no competing interests.

## Authors' contributions

SZ* designed the work, supervised the study, analysed the data and wrote the manuscript. SP carried out polymorphism studies. AAM contributed in the analysis of the data. NDD participated in sample collection, helped with the sequence data analysis and also critical reading of the manuscript. All authors read and approved final manuscript and agree to submission.
